# Small Extracellular Vesicles from Periodontal Ligament Stem Cells Primed by Lipopolysaccharide Regulate Macrophage M1 Polarization via miR-433-3p Targeting TLR2/TLR4/NF-κB

**DOI:** 10.1007/s10753-023-01845-y

**Published:** 2023-06-23

**Authors:** Shuyue Cui, Zijie Zhang, Chen Cheng, Shuai Tang, Mingrui Zhai, Lan Li, Fulan Wei, Gang Ding

**Affiliations:** 1 School of Stomatology, Weifang Medical University, Baotong West Street No. 7166, Weifang, Shandong China; 2 Department of Orthodontics, School and Hospital of Stomatology, Cheeloo College of Medicine, Shandong University & Shandong Key Laboratory of Oral Tissue Regeneration & Shandong Engineering Laboratory for Dental Materials and Oral Tissue Regeneration & Shandong Provincial Clinical Research Center for Oral Diseases, No.44-1 Wenhua Road West, Jinan, Shandong China

**Keywords:** periodontal ligament stem cells, small extracellular vesicles, macrophage, polarization, microRNA.

## Abstract

Lipopolysaccharide (LPS) is regarded as the main pathogenic factor of periodontitis. Mesenchymal stem cell–derived small extracellular vesicles (sEVs) play a key role in a variety of physiological and pathological processes. This study investigated the effects of sEVs derived from periodontal ligament stem cells (PDLSCs) pretreated with LPS on macrophage polarization and the underlying mechanisms. PDLSCs were treated with LPS (1 µg/mL) for 24 h, and sEVs were harvested by gradient centrifugation method. Macrophages were incubated with sEVs for 24 h, followed by examination of the expression profiles of inflammatory and anti-inflammatory cytokines, and polarization markers. Furthermore, microarray analysis, western blot test, and microRNA inhibitor transfection experiments were used to elucidate the molecular signaling pathway responsible for the process. The results showed that sEVs derived from LPS-preconditioning PDLSCs could significantly increase the expression of M1 markers and inflammatory cytokines, whereas decreased the expression of M2 markers and anti-inflammatory cytokines. Mechanistic analysis showed that TLR2/TLR4/NF-κB p65 pathway was involved in M1 polarization of macrophages, and microRNA-433-3p played a role, at least in part, in the course. Collectively, LPS could promote the macrophages into M1 status via TLR2/TLR4/NF-κB p65 signaling pathway partly by sEV-mediated microRNA-433-3p, which could be a potential therapeutic target for periodontitis.

## INTRODUCTION

As an important substance of intercellular communication and rich in a variety of bioactive components, small extracellular vesicles (sEVs) have been proven to be a key paracrine factor for mesenchymal stem cells (MSCs) to play their physiological roles [1]. sEVs are membranous vesicles wrapped by lipid bilayers with a diameter of less than 200 nm, and mainly involved in substance exchange and signal transduction between cells [2–4]. MSC-derived sEVs are involved in body development and differentiation, immune regulation, tumorigenesis and progression, and epigenetic regulation, and have similar functions with their parent cells [5]. In addition, compared with their parent cells, sEVs derived from MSCs have the following advantages: (1) sEVs owned higher safety and lower risk of immune rejection and aneuploidy because of acellular therapy; (2) sEVs are comparatively stable and their contents are not easily degraded during cryopreservation; (3) sEVs can be directly targeted at specific organs and damaged sites; (4) the production cost of sEVs can be controlled, and it is convenient for transportation and preservation [2, 3, 6].

Distributed in almost all tissues, macrophages are a kind of multi-differentiated cells and an important part of the innate immunity of the body, possessing different activation pathways and functional states [7]. After pathogens invade the human body, macrophages could polarize into an M1 state through changes in the body microenvironment, and play a pro-inflammatory role able to produce a large number of inflammatory factors such as interleukin (IL)-1β, IL-6, tumor necrosis factor (TNF)-α, and inducible nitric oxide synthase (iNOS), which are able to kill microorganisms to remove debris and promote an inflammatory response. M2 macrophages exert anti-inflammatory effects and secrete anti-inflammatory cytokines, including IL-10 and transforming growth factor (TGF)-β, which can promote wound healing and tissue repair and regeneration [7–9].

As one of the main components of the cell wall of Gram-negative bacteria, lipopolysaccharide (LPS) plays an important role in innate immune response and is regarded as the main pathogenic factor of periodontal disease, resulting in loss of periodontal tissues, ultimately loss of teeth [10]. In this study, we intend to explore the functions of sEVs derived from periodontal ligament stem cells (PDLSCs) pretreated with LPS on the polarization of macrophages and the underlying mechanisms, so as to find the potential target for periodontal tissue regeneration and the treatment of periodontal disease.

## MATERIAL AND METHODS

### Isolation, Culture, and LPS-Preconditioning of PDLSCs

In this study, normal human impacted third molars or premolars extracted for orthodontic reasons were collected from patients without periodontal disease or dental caries under 23 years old at the Department of Oral and Maxillofacial Surgery, School of Stomatology, Shandong University after the patients gave their informed consent. The methods of isolation, culture, and characterization of PDLSCs were carried out as previously reported [11, 12].

For LPS-preconditioning PDLSCs (LPS pre-PDLSCs), PDLSCs were seeded into a 10-cm cell culture dish to achieve a confluence of 60–70%. After the medium was discarded, the cells were washed three times with phosphate buffer saline (PBS, Biosharp), and treated with α-MEM containing LPS (1 µg/mL, Sigma) at 37℃ in 5% CO_2_ for 24 h.

Extraction of sEVs was performed as previously reported [2, 13]. PDLSCs or LPS pre-PDLSCs were cultured in α-MEM containing 10% sEV-free FBS at 37℃ in 5% CO_2_ for 48 h. sEVs were harvested from the supernatants of PDLSCs or LPS pre-PDLSCs by gradient centrifugation method according to the following steps. The cell supernatants were firstly filtered through a 0.22-µm filter to remove large debris, centrifuged at 12,000 × g for 30 min to remove dead cells, and then centrifuged at 120,000 × g for 70 min to remove cellular debris, and lastly centrifuged at 120,000 × g for 70 min. After this process, the pellets primarily consisted of sEVs, which were re-suspended with PBS for further experiments. sEVs extracted from PDLSCs or LPS pre-PDLSCs are referred to as P-sEVs or LPS pre-sEVs in this study, respectively. The concentrations of sEVs were measured using the BCA protein analysis kit (Solarbio, China). Transmission electron microscopy (TEM; Hitachi HT-7800, Japan) was used to detect the morphology of sEVs. The particle size distribution was identified by ZetaView (Particle Metrix). Western blot was used to detect the expression of CD63 (Abcam), CD9 (Abcam), and ALIX (Wanleibio, China).

### sEV MiRNA Isolation, High-throughput Sequencing, and Quantification

Total RNA was isolated from sEVs using RNAex Pro Reagent according to the manufacturer’s instructions. MiRNA library preparation and sequencing were conducted by using a commercial service (Kegene, China). According to the results of high-throughput sequencing, we used RT-PCR to quantitatively analyze the differentially expressed genes. Reverse-transcript reactions were conducted using the miRNA 1st strand cDNA synthesis kit (AG), and qPCR primers were purchased from Accurate Biology (AG). Relative RNA level was evaluated using the LightCycler-480 system (Roche Diagnostics GmbH, Mannheim, Germany) and SYBR^®^ Green Premix Pro Taq HS qPCR Kit II (AG). U6 was detected as internal controls to quantify the results. The PCR reaction conditions are as follows: 95 ℃ for 30 s, then 40 cycles of 95 ℃ for 5 s, and 60 ℃ for 30 s. The 2^−ΔΔCT^ value was used for comparative quantitation. The information on primer sequences is shown in Table 1.

**Table 1:** Primer Sequences of microRNAs Differentially Expressed in High-Throughput Sequencing Analysis

Primer name	Sense primers (5’-3’)
hsa-miR-654-3p	5′-CCTGCTGACCATCACCTTAAA-3′
hsa-miR-493-3p	5′-TGAAGGTCTACTGTGTGCCA-3′
hsa-miR-411-3p	5′-GTATGTAACACGGTCCACTAAC-3′
hsa-miR-433-3p	5′-ATGATGGGCTCCTCGGTGTAA-3
hsa-miR-379-5p	5′-GGTAGACTATGGAACGTAGGAAA-3′
hsa-miR-6068	5′-GAGTCTCCGGCGGTGGAAA-3′
hsa-miR-494-3p	5′-TGAAACATACACGGGAAACCTC-3′
hsa-miR-21-5p	5′-TAGCTTATCAGACTGATGTTGA-3′

### THP-1 Cell Culture and Treatment

The human monocytic cell line THP-1 cells (purchased from the Cell Bank/Stem Cell Bank, Chinese Academy of Sciences) were cultured in Roswell Park Memorial Institute-1640 medium (BI) supplemented with 10% fetal bovine serum. The cells were grown at a density of 3.0 × 10^5^–6.0 × 10^5^ cells/mL; then, cells were seeded on 6-well plates at 1.0 × 10^6^ cells/well and induced to differentiate by treatment with 50 ng/mL phorbol 12-myristate 13-acetate (PMA; Med Chem Express). After 24 h, the cells were rinsed three times with PBS to remove non-adherent cells. Adherent cells were further incubated with fresh medium containing P-sEVs (10 μg/mL) or LPS pre-sEVs (10 μg/mL) for an additional 24 h.

To trace P-sEVs and LPS pre-sEVs by fluorescent microscopy, they were labeled with PKH67 (Sigma) according to the manufacturer’s instructions and washed in PBS. Then, the PKH67-labeled P-sEVs and LPS pre-sEVs were co-cultured with THP-1 cells at a final concentration of 10 μg/mL and observed at 3 and 12 h to determine the uptake of the labeled sEVs under an inverted phase contrast microscope (Olympus, Japan).

THP-1 cells were seeded on 6-well plates at 1.0 × 10^6^ cells/well with 50 ng/mL PMA. After 24 h, the cells were treated with P-sEVs or LPS pre-sEVs for another 24 h; then, we detected the cell apoptosis as manufacturer’s protocols using an apoptosis kit. Briefly, PDLSCs were collected, centrifugated at 4 ℃ for 5 min, washed twice with PBS, and then 100 μL 1 × Binding Buffer was added into 5.0 × 10^5^ PDLSCs. Next, 5 μL Annexin V-FITC and 10 μL PI Staining Solution were added, mixed gently, and reacted at room temperature in the dark for 15 min. Subsequently, the samples were detected by flow cytometry.

### Quantitative RT-PCR

Total mRNA was separated from THP-1 cells of different treatments using RNAex Pro Reagent and reverse-transcribed into cDNA using an Evo M-MLV Mix Kit with gDNA Clean for qPCR (AG, China) according to the manufacturer’s protocol. The targeted genes comprised GAPDH, IL-6, TNF-α, CD206, TGF-β, CD80, and CD163. The information on primer sequences (BioSune, China) is shown in Table 2. Then, RT-PCR reactions were performed with the LightCycler-480 system and SYBR^®^ Green Premix Pro Taq HS qPCR Kit II. The PCR reaction conditions are the same as described earlier.

**Table 2:** Primer Sequences of Pro-inflammatory and Anti-inflammatory Cytokines and Macrophage Polarization Markers

Primer name	Sense primers (5′-3′)	Antisense primers (3′-5′)
IL-6	TGCAATAACCACCCTGACC	ATTTGCCGAAGAGCCCTCAG
TNF-α	CACTTTGGAGTGATCGGCCC	AGCTTGAGGGTTTGCTACAAC
TGF-β	GCAACAATTCCTGGCGATACC	ATTTCCCCTCCACGGCTCAA
CD80	TGCCTGACCTACTGCTTTGC	AGGGCGTACACTTTCCCTTC
CD163	TCTCTTGGAGGAACAGACAAGG	CCTGCACTGGAATTAGCCCA
CD206	GATTGCAGGGGGCTTATGGG	CGGACATTTGGGTTCGGGAG
GAPDH	GCACCGTCAAGGCTGAGAAC	TGGTGAAGACGCCAGTGGA

### MiRNA Target Prediction and Pathway Enrichment Analysis

Based on the results of high-throughput sequencing, we queried the target gene prediction website (targetscan.org/vert_72/) and found that there are multiple binding sites between miR-433-3p and TLR4.

### Transfection of MiRNA Inhibitors

THP-1 cells were treated with P-sEVs or LPS pre-sEVs, respectively, on a 6-well culture plate, and then were transfected with miR-433-3p inhibitor or negative control at the final concentration of 50 nM using liposome 2000 (Invitrogen). The cells were harvested 48 h after transfection and used in the following experiment.

### Western Blotting

Proteins were collected from treated THP-1 and lysed with RIPA reagent (Solarbio) containing 1% phenylmethanesulfonyl fluoride. After centrifugation at 12,000 rpm at 4 ℃ for 15 min, the total protein concentration was determined using the BCA protein analysis kit. The protein samples were subjected to sodium dodecyl sulfate polyacrylamide gel electrophoresis and incubated with desired antibodies against toll-like receptor (TLR) 4 (Santa Cruz, USA), TLR2, NF-κB p65, p-NF-κB p65 (Wanleibio, China), and GAPDH overnight at 4 ℃. The next day, after rinsing the cells three times with TBST, corresponding secondary antibodies were added and incubated in a shaker at room temperature for 1 h. After incubation, the cells were rinsed three times with TBST and enhanced chemiluminescence was developed chemically.

### Statistical Analysis

Statistical analysis was carried out by using GraphPad Prism8.0 software. All the results are expressed as the mean ± standard deviation (SD) from three independent experiments. Statistical significance was assessed by Student’s *t*-test or analysis of variance; a *p* value less than 0.05 was considered statistically significant.

## RESULTS

### sEV MiRNA Isolation, High-throughput Sequencing, and Quantification

**Fig. 1:**
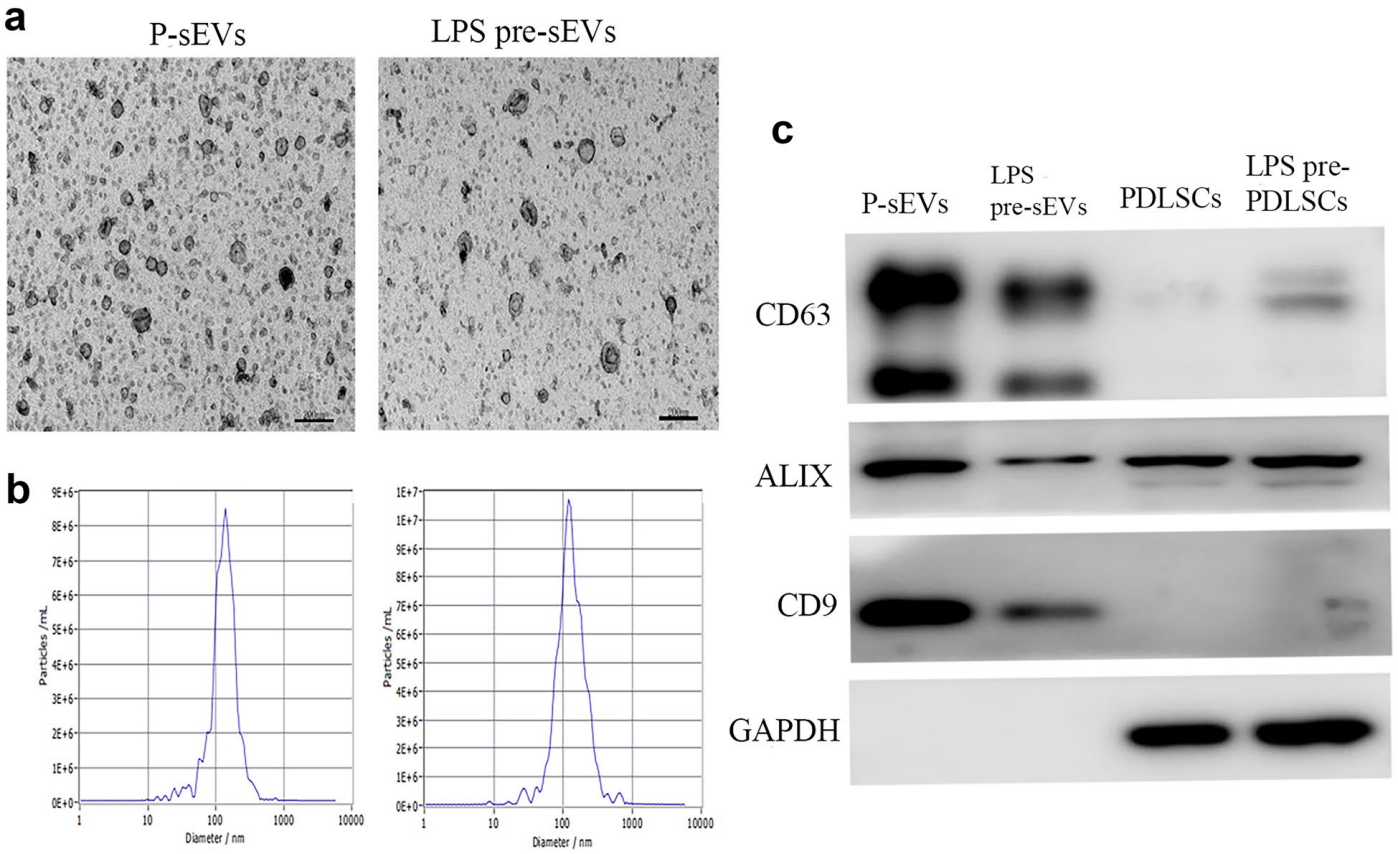
Characterization of P-sEVs and LPS pre-sEVs. **a** The typical morphology of sEVs derived from both PDLSCs and LPS pre-PDLSCs was spherical or goblet bilayer structure under transmission electron microscopy. Scale bar = 200 nm. **b** The particle size of P-sEVs and LPS pre-sEVs reached the peak at 146 nm and 141 nm, respectively. **c** Western blot results showed that P-sEVs and LPS pre-sEVs expressed CD63, ALIX, and CD9, three specific sEV proteins.

PDLSCs, P-sEVs, and LPS pre-sEVs were successfully isolated. The characterization of P-sEVs and LPS pre-sEVs is shown in Fig. 1. The typical morphology of sEVs, derived from PDLSCs or LPS pre-PDLSCs, was spherical or goblet bilayer structure under TEM (Fig. 1a). The particle size of P-sEVs and LPS pre-sEVs reached the peak at 146 nm and 141 nm, respectively (Fig. 1b). Western blot results showed that sEVs from the two groups expressed CD63, ALIX, and CD9, three specific sEV proteins (Fig. 1c).

**Fig. 2:**
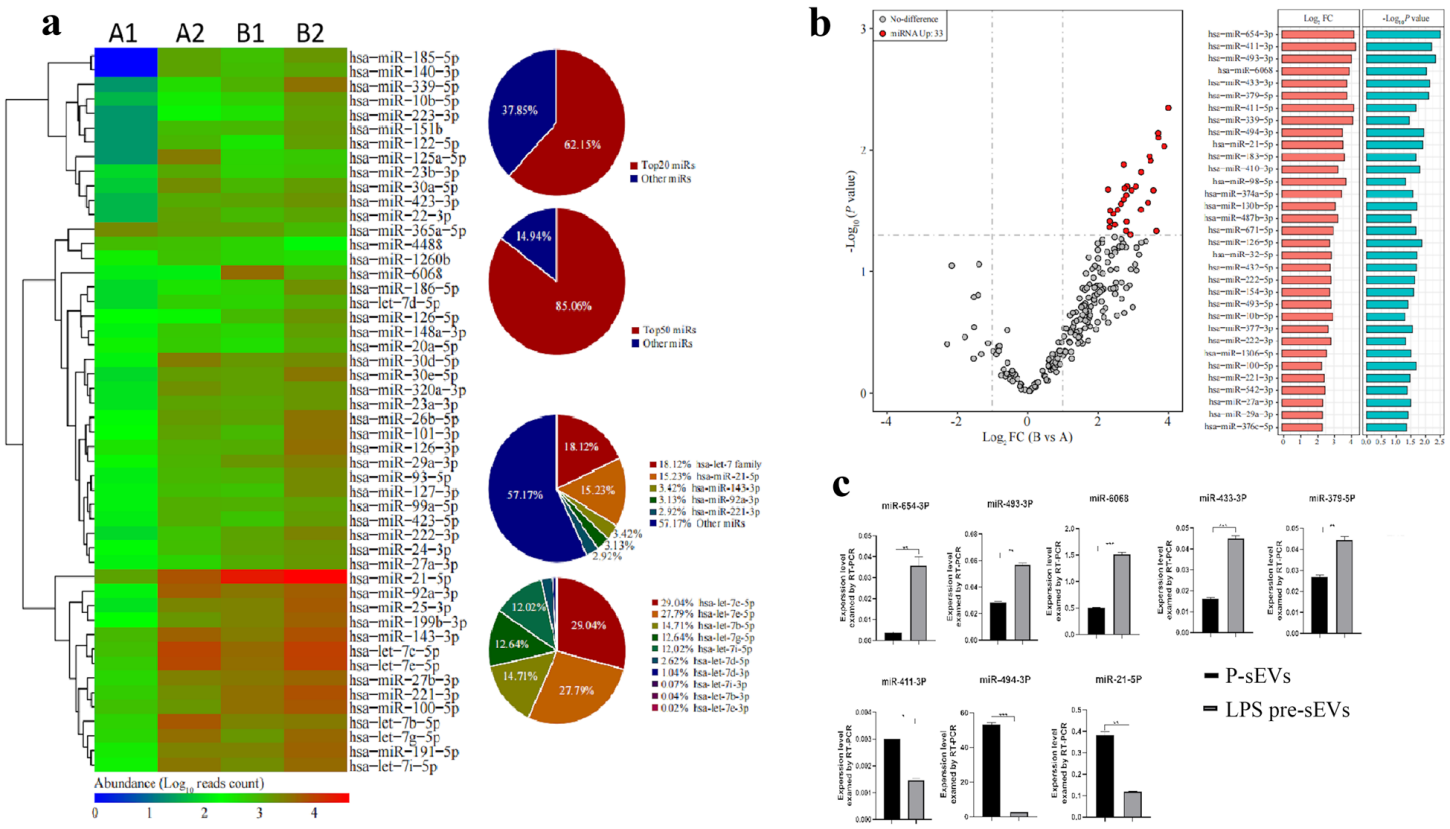
sEV miRNA isolation, high-throughput sequencing, and quantification. **a** The miRNA expression profiles of P-sEVs and LPS pre-sEVs were analyzed by miRNA high-throughput sequencing. Among the observed miRNAs, 20 and 50 kinds of miRNAs with the highest expression accounted for 62.15% and 85.06% of the total miRNAs in P-sEVs and LPS pre-sEVs, respectively. **b** Thirty-three miRNAs were found to express significantly differentially between P-sEVs and LPS pre-sEVs. **c** RT-PCR results showed that compared with P-sEV group, the expression level of miR-654-3P, miR-493-3P, miR-6068, miR-433-3P, and miR-379-5P in LPS pre-sEV group was up-regulated, which was consistent with the results of high-throughput sequencing. However, the RT-PCR results of miR-411-3P, miR-494-3P, and miR-21-5p were unstable and inconsistent or have no statistical difference from those of high-throughput sequencing. A1, A2, B1, and B2 indicate different sEV samples. A1 and A2, not pretreated with LPS. B1 and B2, pretreated with LPS. *p < 0.05. **p < 0.01. ***p < 0.001.

The miRNA expression profiles of P-sEVs and LPS pre-sEVs were analyzed by miRNA high-throughput sequencing. Among the observed miRNAs, 20 and 50 kinds of miRNAs with the highest expression accounted for 62.15% and 85.06% of the total miRNAs in P-sEVs and LPS pre-sEVs, respectively (Fig. 2a). In P-sEVs, the expression of miR-let7 family and miR-21-5p was the highest, accounting for 18.12% and 15.23% of the total miRNA content, respectively. We found 33 miRNAs differentially expressed between P-sEVs and LPS pre-sEVs (Fig. 2b). In order to verify the reliability of the high-throughput sequencing results, eight differentially expressed genes were screened out according to the sequencing results, and were quantitatively analyzed by RT-PCR. The results of RT-PCR showed that compared with the P-sEV group, the expression levels of miR-654-3p, miR-493-3p, miR-6068, miR-433-3p, and miR-379-5p in LPS pre-sEV group were up-regulated statistically, which was consistent with the results of high-throughput sequencing (Fig. 2c). The RT-PCR results of miR-411-3p, miR-494–3, and miR-21-5p were unstable, inconsistent with the results of high-throughput sequencing or not statistically significant (Fig. 2c).

### Cellular Uptake of sEVs and Cell Apoptosis Assay

**Fig. 3:**
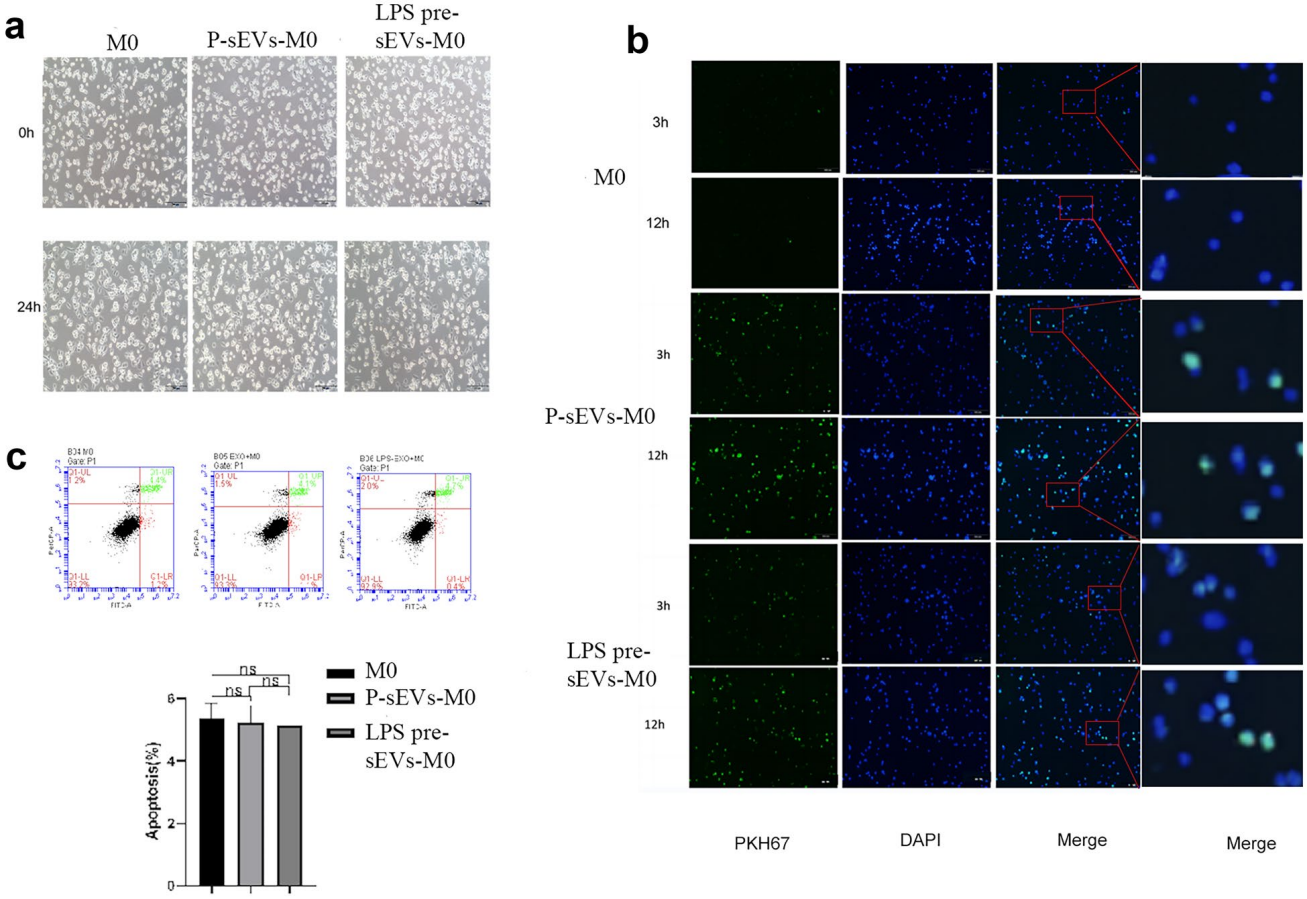
Cellular uptake of sEVs and cell apoptosis assays.** a** After the induction of THP-1 with PMA and the different treatments of sEVs, cells have various shapes, some of the cells were short fusiform, and some were transparent and round under light microscope. Scale bar = 100 μm. **b** The sEVs of different treatments were labeled with PKH67 dye, and then added to the macrophages. Three hours later, the sEVs of the two groups began to be swallowed by macrophages at 3 h, and the fluorescence intensity reached the highest at 12 h. Scale bar = 100 μm. **c** Macrophages co-cultured with sEVs were investigated using apoptosis staining kit, showing P-sEVs or LPS pre-sEVs were not able to induce the apoptosis of macrophages. ns, not significant.

P-sEVs or LPS pre-sEVs were not capable of altering the appearance of the macrophages (Fig. 3a). The sEVs of different groups were labeled with PKH67 dye, and subsequently added to co-culture with the macrophages. We found that the sEVs of the two groups began to be swallowed by macrophages at 3 h post-co-culture, and the fluorescence intensity reached the highest at 12 h (Fig. 3b). P-sEVs or LPS pre-sEVs were not able to induce the apoptosis of macrophages (Fig. 3c).

### LPS Pre-sEVs Converted Macrophage into M1 Phenotype

**Fig. 4:**
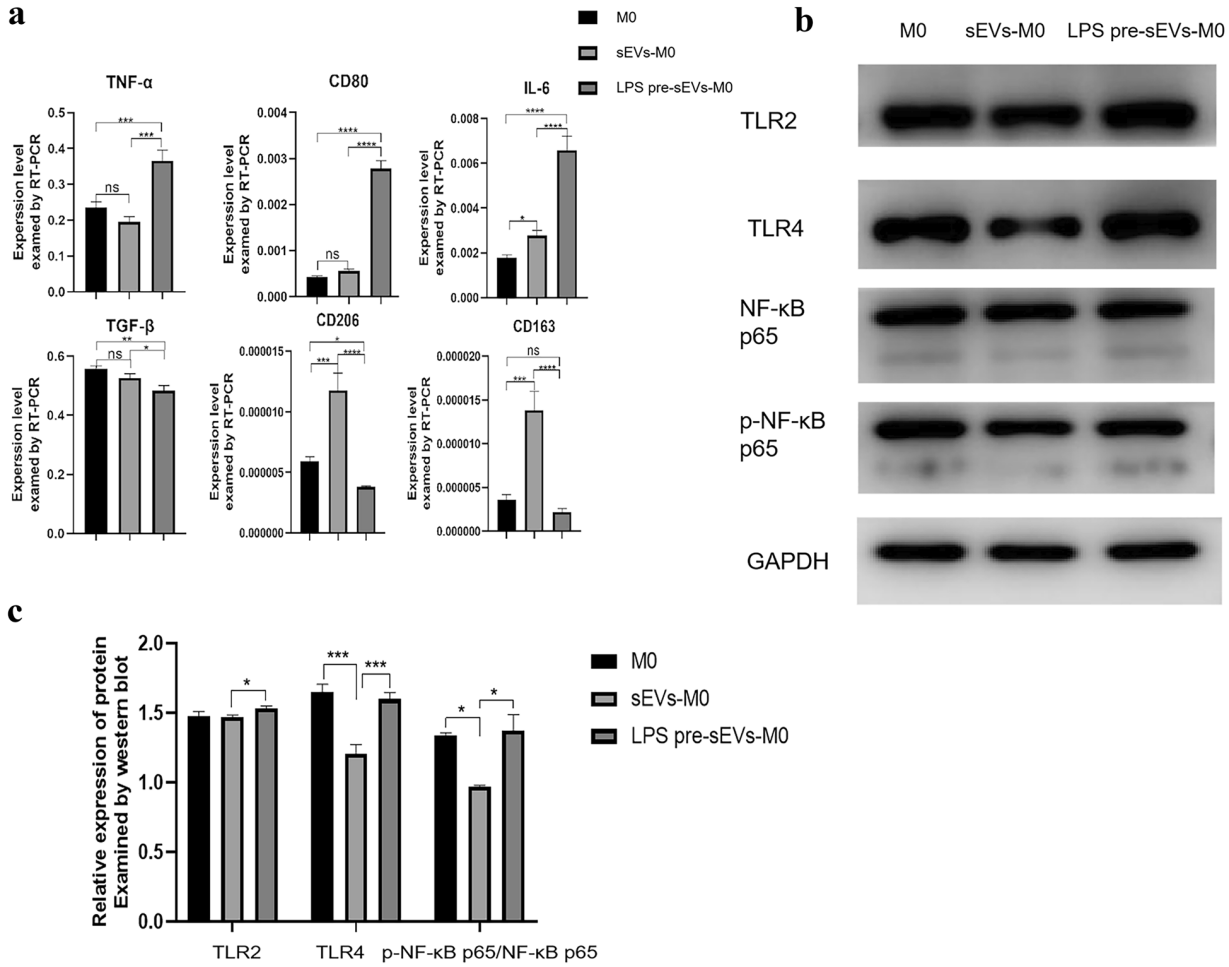
TLR2/TLR4/NF-κB p65 pathway was involved in the macrophage M1 polarization induced by LPS pre-sEVs.** a** After macrophages were co-cultured with LPS pre-sEVs, RT-PCR results showed that LPS pre-sEVs could significantly increase the expression of CD80, M1 marker, and inflammatory cytokines TNF-α and IL-6 on macrophages compared with untreated group, whereas the expression of CD206 and CD163, two M2 markers, and anti-inflammatory cytokine TGF-β was decreased. **b**, **c** The levels of TLR2, TLR4, and p-NF-κB p65 were significantly up-regulated after LPS pre-sEV treatment compared with the control group. *p < 0.05. **p < 0.01. ***p < 0.001. ****p < 0.0001. ns, not significant.

The RT-PCR results showed that LPS pre-sEVs could significantly increase the expression of CD80, M1 marker, and inflammatory cytokines TNF-α and IL-6 on macrophages compared with P-sEV group, whereas the expression of CD206 and CD163, two M2 markers, and anti-inflammatory cytokine TGF-β was decreased. However, P-sEVs could increase the expression of CD206 and CD163 on macrophages. These data suggested that P-sEVs can promote the M2 polarization of macrophages, and LPS pre-sEVs can convert macrophage polarized into M1 phenotype (Fig. 4a). Western blot analyses revealed that the levels of TLR2, TLR4, and p-NF-κB p65 were significantly up-regulated after LPS pre-sEV treatment compared with the control groups (Fig. 4b, c).

**Fig. 5:**
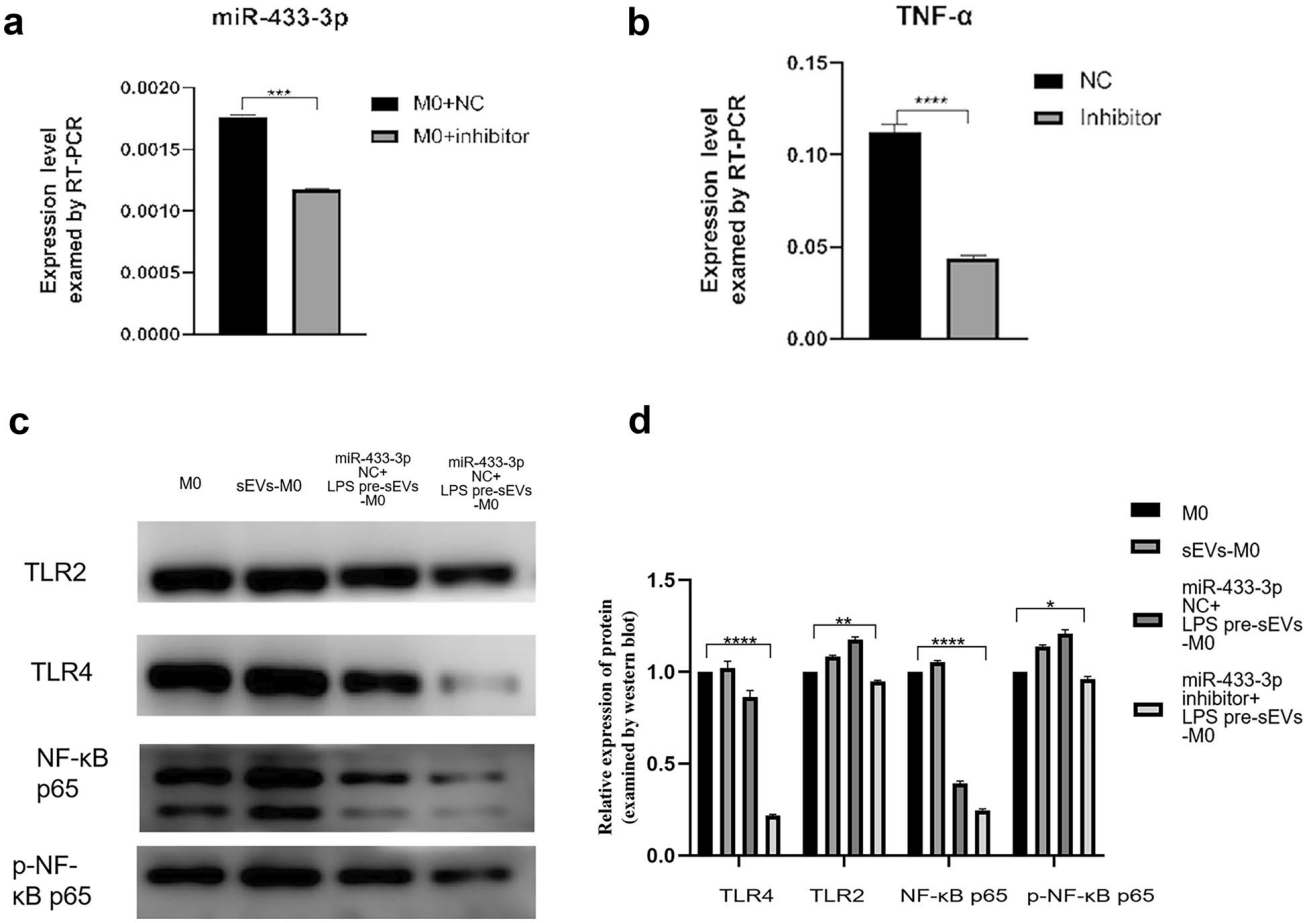
MiR-433-3p is involved, at least in part, in M1 macrophage polarization by LPS pre-sEVs. **a** Transfection of miR-433-3p inhibitor could significantly reduce the expression of miR-433-3p. **b** RT-PCR assays demonstrated that the pro-inflammatory TNF-α was significantly decreased after application of miR-433-3p inhibitor. **c**,** d** MiR-433-3p inhibitor could inhibit the expression of TLR2 and TLR4 and the phosphorylation of NF-κB p65. ***p < 0.001.****p < 0.0001.

To confirm if miR-433-3p was involved in this course, we transfected macrophages with miR-433-3p inhibitor to clarify this circumstance (Fig. 5a). RT-PCR assays found that the expression of a downstream gene of pro-inflammatory TNF-α was significantly decreased in miR-433-3p inhibitor group (Fig. 5b). Furthermore, the results showed that miR-433-3p inhibitor could inhibit TLR2, TLR4, and the phosphorylation of NF-κB p65 (Fig. 5c, d).

## DISCUSSION

When periodontal tissue destruction involves deep tissues, macrophages will release a great deal of inflammatory factors, leading to immune cell infiltration and inflammation, and in turn, infiltration of immune cells, such as memory T cells, will further expand inflammation and produce inflammation factors leading to tissue destruction [14, 15]. The results of RT-PCR showed that after LPS pre-sEV treatment, the expression of TNF-α and IL-6 was up-regulated significantly, whereas the expression of TGF-β and IL-10 was down-regulated. Further RT-PCR analysis found that LPS pre-sEVs increased the expression of CD80 and M1 markers and decreased the expression of CD206 and CD163, two M2 markers, respectively. The opposite results were found in the group of P-sEVs. Collectively, these data indicated that LPS pre-sEVs were able to shift the macrophages into M1 polarization, not M2 polarization. Nowadays, more and more studies have shown that sEVs derived from MSCs can convert the polarization of macrophages toward M2, promote wound healing, and facilitate tissue repair and regeneration [16, 17]. Studies have shown that 100 ng/mL LPS can induce macrophages to polarize toward M2 by sEVs of human umbilical cord MSCs [18], while 1 µg/mL LPS can induce macrophages to polarize toward M1 by sEVs when acting on PDLSCs [13]. It can be seen that LPS at a lower dose may promote stem cells in an anti-inflammation state. These results indicate that sEVs derived from MSCs of different sources or under different culture conditions may have different effects on the polarization of macrophages.

The innate immune response is the first line of defense against pathogenic microbial flora of the host and plays an important role in the immune defense of the body, where TLRs, as cellular transmembrane receptors and pathogen pattern recognition receptors, could recognize pathogenic molecular patterns on the surface of a large number of microorganisms and activate innate immune cells, such as macrophages, through intercellular signaling pathways to stimulate an acquired immune response [19, 20]. In periodontal inflammation, TLR2 and TLR4 in the TLR family can specifically recognize LPS, participate in signal transduction and release inflammatory factors, and play an important role in LPS-induced periodontal tissue destruction [21]. NF-κB is the main signaling pathway activated by TLR2 and TLR4 after LPS recognition. The activated NF-κB signaling pathway can further promote the recruitment and aggregation of inflammatory factors and expand the inflammatory response [22]. Moreover, activation of NF-κB can promote macrophage M1 polarization to enhance inflammation, and TLR4 was activated in inflammatory response by its downstream NF-κB pathway [23].

Collectively, we demonstrated that LPS may convert the PDLSC-induced M2 polarization of macrophages into M1 status partly by sEV-mediated microRNA-433-3p, which could be a potential therapeutic target for periodontitis.

## Data Availability

The data and materials that support the findings of this study are available from the corresponding author upon reasonable request.
